# A totally extraperitoneal approach for endoscopic resection of a congenital prepubic sinus through the pubic symphysis

**DOI:** 10.1186/s40792-021-01245-0

**Published:** 2021-07-15

**Authors:** Masahiro Fukuhara, Shun Onishi, Yusuke Yonemura, Tomoe Sato, Satoshi Tsutsumi, Toshio Bandoh, Tohru Utsunomiya, Genshiro Esumi

**Affiliations:** 1 Department of Pediatric Surgery, Oita Prefectural Hospital, 8-1, Bunyo 2-chome, Oita, 870-8511 Japan; 2 Department of Pediatric Surgery, Kagoshima University, Kagoshima, Japan; 3 Department of Surgery, Kyushu University Beppu Hospital, Beppu, Japan; 4 Department of Gastroenterological Surgery, Oita Prefectural Hospital, Oita, Japan; 5 Department of Pediatric Surgery, University of Occupational and Environmental Health, Kitakyushu, Japan

**Keywords:** Congenital prepubic sinus, Totally extraperitoneal approach, Child

## Abstract

**Background:**

A congenital prepubic sinus (CPS) is a rare congenital anomaly in which a duct remnant extends from the skin opening near the pubic symphysis to various parts and the lesions are mostly located in the preperitoneal space. The totally extraperitoneal (TEP) approach is an operational method that provides a good field of view for the preperitoneal space. We report the CPS through the pubic symphysis in which complete resection was achieved by a TEP approach. TEP approach was minimally invasive and achieved satisfactory cosmetic outcome.

**Case presentation:**

We herein report the case of a 13-year-old boy with a fistula opening near the dorsal penis. He was admitted to our hospital due to fever and lower abdominal pain. Abdominal ultrasonography and computed tomography revealed an abscess inside a fistula lumen on the posterior surface of the rectus abdominis muscles in the midline of the lower abdomen. Under a diagnosis of CPS, which was located in the preperitoneal space, endoscopic resection was performed by a totally extraperitoneal approach. After making an umbilical incision, the rectus abdominis muscle was excised outward to expose the preperitoneal space. A single-port system was placed in the preperitoneal space. Three 5-mm-port trocars were inserted. As the preperitoneal cavity was expanded, a sinus connecting to the pubic symphysis was confirmed. The pubic symphysis did not connect with the bladder. Because the fistula was penetrated with the pubic symphysis, the remaining caudal fistula was removed from the body surface with a small spindle-shaped incision around the fistula opening. Finally, the sinus was completely resected, with confirmation from both the cranial side and dorsal side of the pubic symphysis. We were able to perform complete resection of the CPS with good visibility and without any peritoneal damage. There were no intraoperative complications. His postoperative course was uneventful during the 1-year follow-up.

**Conclusions:**

The TEP approach may be feasible for the resection of a CPS and may allow safe and secure resection due to good visibility, even in pediatric patients.

**Supplementary Information:**

The online version contains supplementary material available at 10.1186/s40792-021-01245-0.

## Background

A congenital prepubic sinus (CPS) is a rare congenital anomaly in which a duct remnant extends from the skin opening near the pubic symphysis to various parts, such as the bladder, umbilicus, pubis and urachal remnants [1]. While a CPS requires complete surgical resection to prevent recurrent infection and the occurrence of malignant tumors, there are wide anatomical variations in its location and morphology [2]. The totally extraperitoneal (TEP) approach is an operational method that provides a good field of view for the preperitoneal space and which is mainly used for inguinal hernia repair [3]. In comparison to the conventional approach, the TEP approach has particularly good visibility within the preperitoneal space, where the CPS is located. In this case, the TEP approach facilitated the minimally invasive complete resection of the CPS. We present our TEP approach for the resection of a CPS through the pubic symphysis.

## Case presentation

**Fig. 1:**
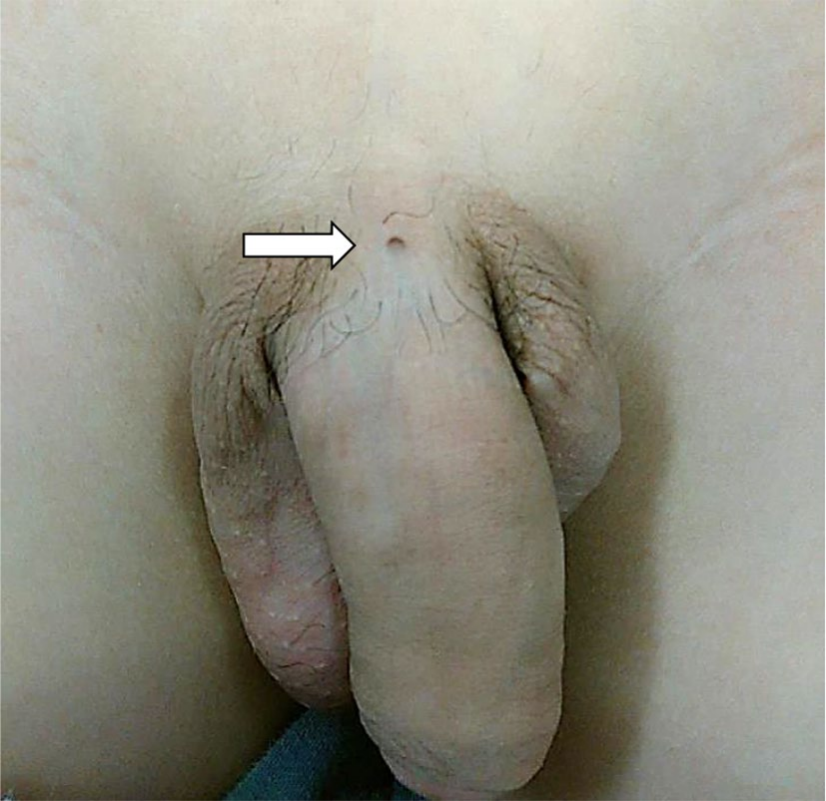
The fistula opening near the dorsal penis (arrow)

**Fig. 2:**
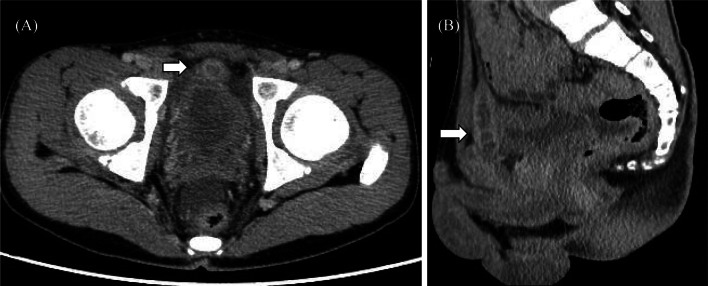
Computed tomography revealed an abscess inside a fistula lumen on the posterior surface of the rectus abdominis muscles in the midline of the lower abdomen (arrows) (**A**: axial **B**: sagittal)

A 13-year-old boy with an asymptomatic fistula opening near the dorsal penis was admitted to our hospital due to fever and lower abdominal pain (Fig. 1). The fistula was recognized at least several months, and it was the first time of the appearance of symptoms. His body temperature was 38.0℃ and hematologic examination revealed a white blood cell count of 8200/μL with neutrophil predominance, a C-reactive protein (CRP) level of 6.15 mg/dL, and high inflammatory response. Abdominal ultrasonography and computed tomography revealed an abscess inside a fistula lumen on the posterior surface of the rectus abdominis muscles in the midline of the lower abdomen (Fig. 2). The size of abscess was 50 × 10 × 10 mm. These fistulas seemed to be connected across the pubis. He was diagnosed with CPS, and surgery was scheduled after treatment of the inflammation with antibiotics. The patient’s clinical symptoms improved after antibiotics administration for 18 days with cefmetazole (1 g/8 h).

**Fig. 3:**
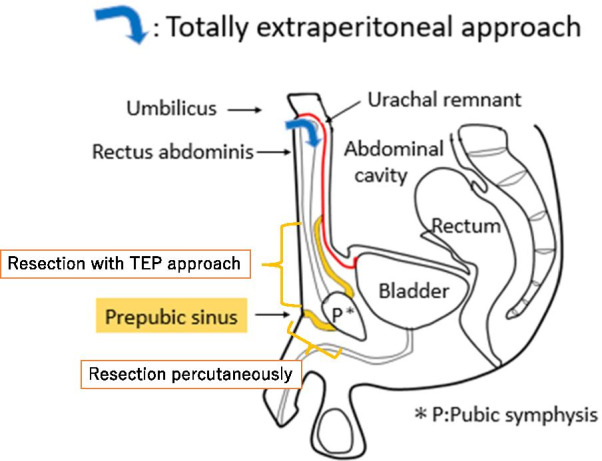
A schema of the TEP approach

**Fig. 4:**
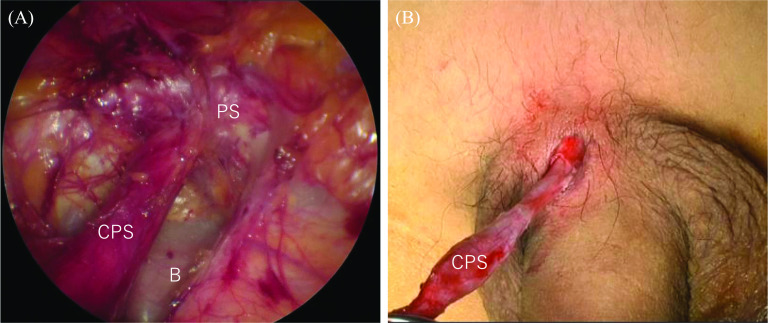
**A** A sinus connecting to the pubic symphysis was confirmed and revealed through with the pubic symphysis. **B** The remaining caudal fistula was removed percutaneously with a small spindle-shaped incision around the fistula opening. *CPS* congenital prepubic sinus, *PS* pubic symphysis, *B* bladder

Under general anesthesia, the patient was placed in the supine position. Initially, we made a Y-shaped incision, a semicircular incision in the natural skin crease immediately below the umbilicus, with a longitudinal incision below the umbilicus. After making the umbilical incision, the rectus abdominis muscle was excised outward to expose the preperitoneal space. The E‧Z Access/LAP-PROTECTOR mini-type device (Hakko Co., Ltd., Tokyo, Japan), a single-port system, was placed in the preperitoneal space (Fig. 3). The preperitoneal space was established with 8-mm Hg carbon dioxide inflation (5 L/min). Three 5-mm-port trocars were inserted through the device plate using a 30° laparoscope. Inflammatory adhesions appeared in the preperitoneal space. After dissecting these adhesions, a sinus connecting to the pubic symphysis was confirmed (Fig. 4A). The pubic symphysis did not connect with the bladder. Because the fistula passed through the pubic symphysis, the remaining caudal fistula was removed percutaneously with a small spindle-shaped incision around the fistula opening (Fig. 4B). Finally, the sinus was completely resected, with confirmation from both the cranial side and dorsal side of the pubic symphysis. The total procedure time was 197 min. There were no intraoperative complications. The postoperative course was uneventful. The patient was discharged on postoperative day 5. No recurrence has been observed during 1 year of follow-up.

## Discussion

CPS is rare congenital anomalies of the urinary tract, which is often associated with purulent discharge from a midline opening overlying the pubis. Sakaguchi et al. reported a review of 44 cases including 23 males and 21 females, aged 1 month to 22 years [4]. According to the report, CPS most commonly presents in infancy with discharge from a midline opening between the dorsal penile root/clitoris to the suprapubic region. Most of CPS passed above the pubis or through the pubic symphysis. Conventional surgical procedures include transverse incision in the lower abdomen (Pfannenstiel incision) or a step ladder incision is needed to achieve complete resection [5, 6].

This is the first report to describe the application of the TEP approach for the resection of a CPS in a child. The TEP approach provided good visibility in the preperitoneal space where the main locus of the lesion was located. We could perform the operation with confirmation of the anatomical position of the prepubic sinus through the pubic symphysis due to the good visibility.

CPSs are classified into 3 types: high type, with the sinus extending toward the urachal remnant; middle type, with the sinus extending toward the bladder; and low type, with the sinus extending toward the urethra [2]. Most CPSs are high or middle type [4].This means that the lesions are mostly located in the preperitoneal space. The present case was a high type lesion because the sinus passed through the pubic symphysis and extended toward the urachal remnant. The TEP approach might be suitable for high and middle-type cases located in the preperitoneal space.

In this case, the main locus of the lesion was in the preperitoneal space. The CPS extends from the skin opening near the pubic symphysis to various sites, including the bladder, umbilicus, pubic bone and urethral remnants. In addition, it has anatomical variations: passing through the pubic symphysis or crossing above the pubic symphysis [1].

The use of the TEP approach provided good visibility to confirm the anatomical location of the prepubic sinus through the pubic symphysis. A CPS requires complete surgical resection to prevent recurrent infection and the occurrence of malignant tumors. As far as we searched none of the patients who underwent complete resection had recurrence and complete excision of the CPS in the reported cases was curative. Because it is difficult to secure a sufficient operative field by minimal incision around the fistula opening alone, the wounds get bigger and more numerous. As the sinus passed through the pubic symphysis in this case, complete resection by the conventional approach would have been difficult. In the present case, the laparoscopic TEP approach allowed for minimally invasive surgery of the pediatric patient (Additional file 1).

There are some technical considerations when using the TEP approach in the treatment of children. When dissecting the preperitoneal space, it is important to avoid bleeding from the inferior epigastric vessels and to avoid injuring the peritoneum. Bleeding or peritoneal injury reduce visibility in the limited working space of the child. The TEP approach is often performed with telescopic dissection or balloon dissection [7, 8]. We use telescopic dissection under direct visualization. Because the operation in the small cavity of children can be performed more safely under better visualization in comparison to that achieved with a preperitoneal dissecting balloon. We could perform the operation under telescopic dissection with a good field of view, without any vascular or peritoneal injury.

An extraperitoneal approach without the use of endoscopic resection has been reported to be useful for urachal remnants; the procedure was applied without peritoneal damage and minimized the incidence of adhesive small bowel obstruction [9]. Similar to the treatment of CPSs, the TEP approach might be useful and feasible for the treatment of urachal remnants located in the preperitoneal space.

## Conclusion

Due to good visibility, the TEP approach allowed safe and secure resection of a CPS in a pediatric patient.

## Supplementary Information

**Additional file 1. **The operative procedure of the TEP approach for the endoscopic resection of a CPS through the pubic symphysis.

## Data Availability

The datasets supporting the conclusions of this article are included within the article.

## Abbreviations

CPS: Congenital prepubic sinus
TEP: Totally extraperitoneal

## References

[1] Campbell J, Beasley S, Mcmullin N, Hutson JM (1987). Congenital prepubic sinus: possible variant of dorsal urethral duplication (Stephens type 2). J Urol.

[2] Soares-oliveira M, Julia V, Aparicio LG, Morales L (2002). Congenital prepubic sinus. J Pediatr Surg.

[3] McKernan JB, Laws HL (1993). Laparoscopic repair of inguinal hernias using a totally extraperitoneal prosthetic approach. Surg Endosc.

[4] Sakaguchi T, Hamada Y, Nakamura Y (2016). Congenital prepubic sinus: a case report and review of the literature. J Pediatric Surg Case Rep.

[5] Daher P, Diab N, Moussa Ch (1994). Congenital prepubic sinus. Eur J Pediatr Surg.

[6] Yamada K, Kanamori Y, Hideaki T (2013). Congenital prepubic sinus associated with a urachal remnant: report of a case. Surg Today.

[7] Tastaldi L, Bencsath K, Alaedeen D (2019). Telescopic dissection versus balloon dissection for laparoscopic totally extraperitoneal inguinal hernia repair(TEP): a registry-based randomized controlled trial. Hernia.

[8] Bittner R, Arregui ME, Bisgaard T, Dudai M (2015). Guidelines for laparoscopic (TAPP) and endoscopic (TEP) treatment of inguinal hernia [International Endohernia Society (IEHS)]. Surg Endosc.

[9] Imaizumi T, Urano M, Tanaka N (2017). A surgical technique for urachal excision: transumbilical extraperitoneal tunneling. J Jpn Soc Pediatr Surg.

